# Patient and caregiver perceptions of oxygen therapy in facility-based settings for acute hypoxemic conditions: a scoping review

**DOI:** 10.7189/jogh.15.04084

**Published:** 2025-04-25

**Authors:** Marcello Britto, Ayobami A Bakare, Hamish Graham, Carina King

**Affiliations:** 1 Department of Global Public Health, Karolinska Institutet, Stockholm, Sweden; 2 Access to Medicine Foundation, Amsterdam, the Netherlands; 3 Department of Community Medicine, University College Hospital Ibadan, Ibadan, Nigeria; 4 Melbourne Children’s Global Health, Murdoch Children’s Research Institute, University of Melbourne, Melbourne, Victoria, Australia; 5 Royal Children’s Hospital, Melbourne, Victoria, Australia; 6 Department of Paediatrics, University College Hospital Ibadan, Ibadan, Nigeria

## Abstract

**Background:**

Access to oxygen therapy is essential for ensuring a functioning health care system. Despite its widespread application across multiple patient groups and all ages, there is a lack of understanding about community perceptions and experiences while receiving oxygen therapy for acute conditions. We aimed to understand patient and caregiver perceptions of oxygen therapy in facility-based settings for acute hypoxemic conditions.

**Methods:**

We conducted a scoping review. We searched Medline, Embase, and Web of Science (26 February 2024) for original studies published since 2000 relating to patient or caregiver perceptions and experiences of oxygen for an acute medical need in health facility settings. We used an adapted theoretical framework of acceptability to extract and structure the findings.

**Results:**

Searches returned 10 425 unique records, and 25 articles were included. 20 were from high-income countries, and 18 were qualitative studies. The results showed that patient and caregiver attitudes and feelings about oxygen therapy are strongly influenced by perceived effectiveness, which was almost universally positive. Patients and their caregivers suffer different types of burdens, and these are greater for more advanced respiratory therapies than for simple oxygen therapy. Patient and caregiver understanding of oxygen therapy was low, presenting an opportunity for improved communication. Opportunity costs were highest in caregivers of neonates, who were often separated from their infants for long periods, and out-of-pocket costs were a major consideration in low- and middle-income countries.

**Conclusions:**

In this scoping review, we found distinctions in caregiver and patient burden, and their perspectives of oxygen varied between country income. Intervention coherence – the extent to which the patient and their caregivers understand the treatment – was singled out as the key policy area for improvement. Educational campaigns, like those implemented for previous public health emergencies, could lead to increased public knowledge, and thus acceptability, of oxygen therapy.

Oxygen therapy is one of the most commonly indicated hospital treatments [1] and is defined by the World Health Organization (WHO) as an essential medicine [2]. It is a safe and effective treatment for hypoxemia, a common complication of a range of primary lung and other systemic illnesses. It is a critical component of surgery and anaesthesia [1,3-5]. Hypoxemia is defined as low levels of oxygen in the blood [5]. Clinically, this is usually characterised by a pulse oximetry measurement of peripheral haemoglobin oxygen saturation, where the normal range at sea level is between 95–100% [5]. Hypoxemia has a wide range of causes, including respiratory infections such as pneumonia and COVID-19, chronic respiratory diseases such as chronic obstructive pulmonary disease (COPD), non-respiratory infections such as sepsis and severe malaria, cardiovascular conditions such as heart failure, trauma such as pneumothorax caused by blunt force, and neonatal conditions such as congenital heart disease [4-6].

The recent COVID-19 pandemic acutely increased the demand for medical oxygen and highlighted longstanding global inequity in its availability [7,8]. One study investigating critically ill patients in 10 African countries reported a higher mortality rate than from studies done in Asia, Europe, North America, and South America [9]. The increased mortality was associated with insufficient access to critical care resources, namely oxygen, with half of the patients dying from COVID-19 in intensive care units not receiving medical oxygen therapy [9]. As oxygen therapy is essential to patients across all age groups and a wide variety of conditions, improving access is crucial to achieving six of the eight targets for the third Sustainable Development Goal. On 30 May 2023, the 76th World Health Assembly adopted a resolution urging member states to increase global access to medical oxygen and proposing 20 recommendations for achieving this goal [10]. One of the recommendations specifically addressed public perceptions of oxygen, stating that governments should ‘increase public understanding of hypoxaemia and its consequences and to build confidence in health system capacities to meet medical oxygen needs’ [10].

The tolerance, adherence, and acceptability of long-term oxygen therapy has been well documented – but mainly in home oxygen for obstructive sleep apnoea and COPD in high-income countries [11-14]. However, the same cannot be said for the perspective of the patient undergoing oxygen therapy for an acute medical condition. Some studies have indicated that issues such as fear of oxygen therapy exist, but we know little about the scale or causes of this fear or of other possible misconceptions [15,16]. Indeed, despite oxygen therapy being commonly used for decades, few studies have explored the experience of patients and their caregivers receiving oxygen in hospital settings [17]. This lack of data are troubling, considering that patient adherence is an important factor in ensuring that oxygen therapy is effective [18,19], and patient fear, mistrust, and incomprehension often delay care-seeking and can lead to worse health outcomes [20-22]. We note that it is particularly important to include caregiver perspectives, as infants, children, youth, and other at-risk populations are often reliant on their caregivers to access medical care when the need arises. We use the term caregiver to indicate an informal, untrained individual such as a family member or friend providing unpaid care and support to a patient.

Recognising these challenges and the importance of patient-centred care in policy, practice, and research [23-25], in this scoping review, we focused on the acceptability of oxygen therapy to patients and their caregivers in facility-based settings. We aimed to understand patients’ experiences regarding oxygen therapy for acute conditions in facility-based settings and that of their caregivers, as well as identify the components that make the treatment acceptable to them.

## METHODS

### Study design

We conducted a scoping review of academic literature using the method outlined by Arksey and O’Malley [26]. A scoping review was chosen to address the aim as this method addresses broader topics than systematic reviews and those with limited pre-existing information. While reviews have focussed on patient perspectives and experience with long-term oxygen therapy, the same has not been done for facility-based oxygen therapy for acute conditions. We, therefore, focused our scoping review on this population to characterise currently published studies and identify key knowledge gaps to guide future research on this topic. In this review, we followed the Preferred Reporting Items for Systematic Reviews and Meta-Analyses Extension for Scoping Reviews (PRISMA-ScR) checklist and statement [27].

### Search strategy

We performed a literature search in Medline, Embase, and Web of Science. The initial search was conducted on 27 December 2022 and then re-run on 26 February 2024.

We developed the search strategy in Medline (Ovid) in collaboration with librarians at the Karolinska Institute University Library. For each search concept, we identified MeSH terms and free text terms. The search was then translated into the other databases. The main search terms were: (‘oxygen’ OR ‘oxygen saturation’) AND (‘patient experience’ OR ‘compliance’ OR ‘uptake’) AND (‘community’ OR ‘caregiver’ OR ‘family’ OR ‘parent’ OR ‘patient’).

We restricted the search to English, French, and Spanish language articles published since 2000. Another librarian peer reviewed the search strategies before execution. De-duplication was done using the method described by Bramer et al. [28] to reduce the results from 18 170 to 10 428. We added one final, extra step to compare digital object identifiers (Figure S1 in the **Online Supplementary Document**).

### Inclusion and exclusion criteria

We focused on oxygen therapy for acute medical care in hospital settings. Therefore, we excluded studies focusing on oxygen used in the home or for chronic conditions such as sleep apnoea or COPD, oxygen as part of life-support or palliative care, and oxygen use during surgery. To highlight the community perspective on oxygen therapy, health care professionals and mass media perspectives were excluded. We expanded the perspective of the patient undergoing oxygen therapy to include relatives and caregivers of these patients to properly encompass wider community perspectives, and it is particularly relevant in the case of children. Clinical outcomes such as adherence to the treatment were not included as patient perspectives. We excluded non-original research, including reviews, grey literature and unpublished studies (**Table 1**).

**Table 1:** Study selection criteria

Inclusion
Oxygen provision for acute conditions
Facility-based
Patient, caregiver, or community perspective
Any form of inhaled oxygen therapy (including hyperbaric)
Original research of any study design
Peer-reviewed work
**Exclusion**
Care-home/palliative care
Life-support (including mechanical ventilation)
Surgery
Animal study
Not published in English, French or Spanish
Published before 2000

### Screening

We imported 10 428 search results to Covidence (Veritas Health Innovation, Melbourne, Victoria, Australia) for screening, which also filtered for duplicates – excluding three records. The results were first screened according to the title, and non-relevant studies were discarded. The first 174 papers were double screened independently (MB and CK) to clarify our inclusion and exclusion criteria. Conflicts were resolved through discussion, and the inclusion and exclusion criteria were refined to better exclude non-relevant studies. This was followed by abstract and full-text screening by MB, with any uncertainties discussed and decided by MB and CK.

### Data charting and analysis

We performed data extraction in Microsoft Excel (Microsoft Corporation, Redmond, Washington, USA), with both descriptive and methodological indicators extracted. We used the theoretical framework of acceptability (TFA) designed by Sekhon et al. [29] to structure the analysis, defining acceptability as ‘a multi-faceted construct that reflects the extent to which people delivering or receiving a health care intervention consider it to be appropriate, based on anticipated or experienced cognitive and emotional responses to the intervention’ [29]. The TFA has seven domains – affective attitude, burden, ethicality, intervention coherence, opportunity cost, perceived effectiveness and self-efficacy. After completing data charting, we adapted the framework to remove domains which were not found in the literature and included an overarching theme of context. The TFA also differentiates between three separate temporal perspectives of acceptability – prior acceptability (pre-intervention), concurrent acceptability (during intervention delivery), and retrospective acceptability (post-intervention).

We used the TFA to guide the analysis of the included studies, using directed content analysis [30]. While the overall aim was to understand patient and caregiver perceptions of oxygen therapy in facility-based settings for acute conditions, further areas of interest included investigating differences between patient and caregiver experiences and if medical oxygen is understood and experienced differently across geographies, patient groups, and care settings.

### Quality appraisal

We used the Critical Appraisal Skills Programme (CASP, Oxford, UK) checklist to evaluate the quality of each included article [31]. All articles were retained, regardless of quality, to provide a full picture of the published academic literature on this topic (Table S1 in the **Online Supplementary Document**).

## RESULTS

**Figure 1:**
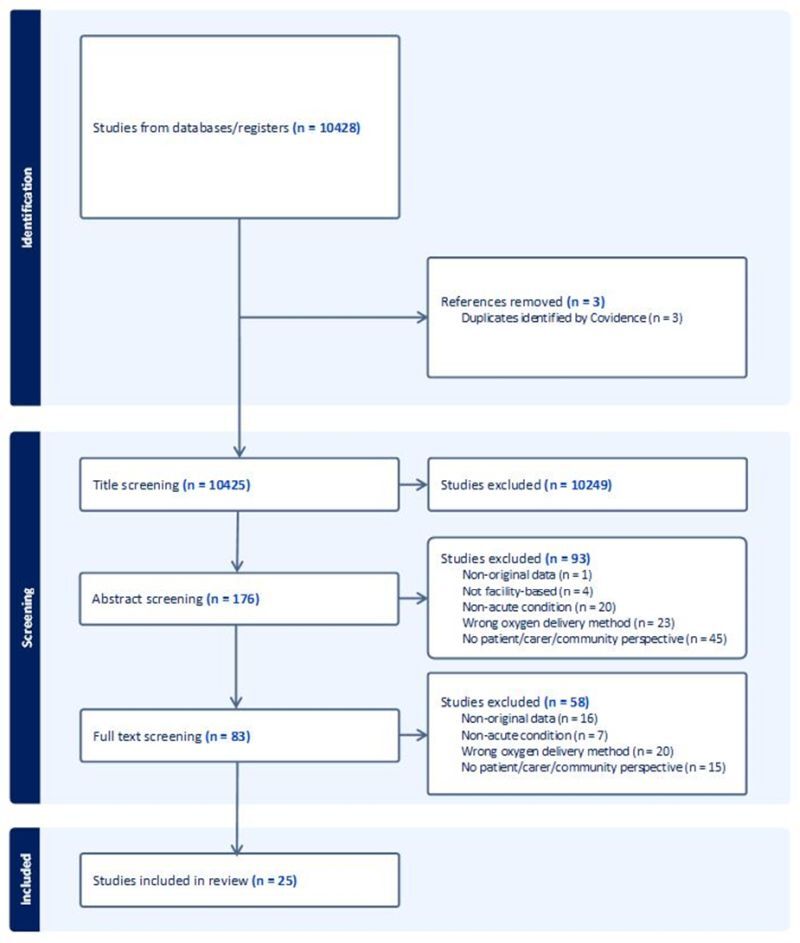
PRISMA diagram for study screening and inclusion.

Following title screening, we retained 176 of the 10 425 results. Out of these, 83 were retrieved based on the abstract. A second researcher independently reviewed the excluded articles. Those remaining were then assessed for eligibility by reading the full text, resulting in 25 articles being included. The most common reason for exclusion after full text evaluation was the wrong oxygen delivery method (**Figure 1**; Table S2 in the **Online Supplementary Document**).

Overall, the included articles scored well in the quality appraisal, reflecting a high standard of methodological rigor, with one notable exception [32]. Qualitative studies scored worst on the relationship between researcher and participants being considered, and quantitative studies scored worst on the blinding. The quality appraisal is available in Table S1 in the **Online Supplementary Document**.

### Publication description

**Figure 2:**
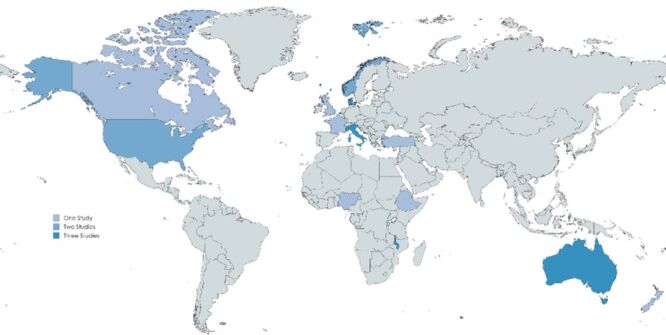
Included studies charted by geographical setting.

Using the World Bank country classification by income group [33], of the 13 countries represented, nine were high-income, one upper-middle income, one lower-middle income, and two were low-income countries (**Figure 2**, **Table 2**). No studies were found from Latin America, the Caribbean, or the South or East Asian regions, and four countries (Italy, Denmark, Australia, and Malawi) accounted for nearly half the studies (n/N = 12/25, 48%).

**Table 2:** Selected indicators of included studies

Study	Title	Study type	Country	Income status	Patient population (x̄ age)	Participants (n)	Healthcare setting	Condition	Oxygen delivery method
Adeoti et al. 2022 [34]	Misconception on oxygen administration among patients and their caregivers in Ado Ekiti, Nigeria	Descriptive cross-sectional study	Nigeria	Lower middle-income	Adults (45.8 y)	873	NA	NA	General oxygen therapy
Ayhan et al. 2009 [18]	Comparison of two different oxygen delivery methods in the early postoperative period: randomised trial	Randomised trial	Turkey	Upper middle-income	Adults (44.0 y)	106	PACU	Post-thyroidectomy	Oxygen mask and nasal cannula
Beckert et al. 2020 [35]	What can we learn from patients to improve their non-invasive ventilation experience? ‘It was unpleasant; if I was offered it again, I would do what I was told’	Qualitative descriptive	New Zealand	High-income	Adults (69.2 y)	15	Respiratory ward	COPD exacerbation	Non-invasive ventilation
Bitterman et al. 2016 [32]	Design and Human Factors of Therapeutic Hyperbaric Chambers	Mixed methods	NA	NA	NA	NA	NA	NA	Hyperbaric chamber
Brugnolli et al. 2023 [36]	Qualitative study of COVID-19 patient experiences with non-invasive ventilation and pronation: strategies to enhance treatment adherence	Qualitative descriptive	Italy	High-income	Adults (63.1 y)	20	ICU of first-level hospital	COVID-19	Helmet CPAP
Cervantes et al. 2011 [37]	The experience of mothers whose very low-birth-weight infant requires the delivery of supplemental oxygen in the neonatal intensive care unit	Qualitative descriptive	Canada	High-income	Adults (32.8 y)	11	NICU	First-time NICU mothers of VLBW infants requiring oxygen delivery for at least seven days	Various (intubation most common)
Cirit Ekiz et al. 2022 [38]	Comparison of full-face and oronasal mask effectiveness in hypercapnic respiratory failure patients with non-invasive mechanical ventilation	Randomised trial	NA	NA	Adults (69 y)	50	Primary and secondary care centres	Hypercapnic respiratory failure	Full face mask and oronasal mask
Constantin et al. 2009 [39]	Impact of sophrology on non-invasive ventilation tolerance in patients with acute respiratory failure	Randomised trial	France	High-income	Adults (50 y)	27	Adult ICU	Acute respiratory failure	Non-invasive ventilation
Dimech et al. 2012 [40]	Critical care patients' experience of the helmet continuous positive airway pressure	Qualitative descriptive	UK	High-income	Adults	6	Critical care unit	Acute respiratory failure	Helmet CPAP
Eastwood et al. 2009 [41]	Patients' and nurses' perspectives on oxygen therapy: a qualitative study	Qualitative descriptive	Australia	High-income	Adults (68 y)	37	ICU	Various (cardio-thoracic patients most common)	Low-flow oxygen therapy (NPO, NP, FM)
Foster et al. 2008 [42]	Parental stress and satisfaction in the non-tertiary special care nursery	Randomised trial	Australia	High-income	Adults	42	Non-tertiary special care unit	Parents of infants requiring oxygen therapy	CPAP and headbox
Gebre et al. 2022 [43]	Perception and experience of clinicians and caregivers in treating childhood severe pneumonia and hypoxemia using bubble continuous positive airway pressure in Ethiopian tertiary and general hospitals	Qualitative descriptive	Ethiopia	Low-income	Adults	15	Two tertiary and two general hospitals	Caregivers/clinicians to children with severe pneumonia and hypoxemia	Bubble CPAP
Gondwe et al. 2017 [44]	Experiences of caregivers of infants who have been on bubble continuous positive airway pressure at Queen Elizabeth Central Hospital, Malawi: A descriptive qualitative study	Qualitative descriptive	Malawi	Low-income	Adults	12	Nursery unit and paediatric nursery ward	Parents of infants requiring bubble CPAP	Bubble CPAP
Hansen et al. 2018 [45]	Automated oxygen control with O2matic during admission with exacerbation of COPD	Randomised trial	Denmark	High-income	Adults (72.4 y)	19	Pulmonary centre	COPD exacerbation	Automated oxygen delivery by O2matic
Klingenberg et al. 2014 [46]	Patient comfort during treatment with heated humidified high flow nasal cannula *vs*. nasal continuous positive airway pressure: a randomised crossover trial	Randomised trial	Norway	High-income	Infants (29.3 weeks)	20	Neonatal unit	Mild respiratory illness	Heated humidified high flow nasal cannula and nasal CPAP
Lucchini et al. 2019 [47]	How different helmet fixing options could affect patients' pain experience during helmet-continuous positive airway pressure	Non-randomised crossover study	Italy	High-income	Adults (60 y)	20	Referral ECMO centre ICU	Respiratory failure	Helmet CPAP
Lucchini et al. 2024 [48]	Patients' recollections of helmet-CPAP treatment during COVID-19 pandemic: A qualitative study	Qualitative descriptive	Italy	High-income	Adults (50 y)	24	ICU	COVID-19	Helmet CPAP
McCormick et al. 2022 [49]	Exploring Patient Experience with Non-invasive Ventilation: A Human-Centered Design Analysis to Inform Planning for Better Tolerance	Qualitative descriptive	USA	High-income	Adults	16	Primary care centre	COPD exacerbation	Non-invasive ventilation
Peeler et al. 2015 [50]	The experiences of parents and nurses of hospitalised infants requiring oxygen therapy for severe bronchiolitis: A phenomenological study	Qualitative descriptive	Australia	High-income	Adults	12	Tertiary paediatric hospital	Parents of children with severe bronchiolitis	Headbox oxygen and high-flow nasal prong oxygen therapy
Peterson et al. 2023 [51]	Symptoms in Patients Receiving Non-invasive Ventilation in the Intensive Care Unit	Descriptive cross-sectional study	USA	High-income	Adults (68 y)	114	ICU at the academic medical centre	Respiratory failure	Bi-level positive airway pressure
Sandau et al. 2022 [52]	Patients’ Perspective on Automated Oxygen Administration during Hospitalization for Acute Exacerbation of Chronic Obstructive Pulmonary Disease: A Qualitative Study Nested in a Randomized Controlled Trial	Qualitative descriptive	Denmark	High-income	Adults	18	Respiratory ward	COPD exacerbation	Automatic oxygen administrator (nasal cannula)
Sandau et al. 2023 [53]	Automated Oxygen Administration Alleviates Dyspnea in Patients Admitted with Acute Exacerbation of COPD: A Randomized Controlled Trial	Randomised controlled trial	Denmark	High-income	Adults (75.3 y)	157	Respiratory ward	COPD exacerbation	Automated oxygen delivery by O2matic
Sessions et al. 2020 [54]	Focus group discussions on low-flow oxygen and bubble CPAP treatments among mothers of young children in Malawi: a CPAP IMPACT sub study	Qualitative descriptive	Malawi	Low-income	Teenagers and adults (26.7 y)	54	NA	Mothers of children with severe pneumonia	Low-flow oxygen and bubble CPAP
Stevenson et al. 2015 [15]	Fear of oxygen therapy for children in Malawi	Qualitative descriptive	Malawi	Low-income	Adults (38.4 y)	97	NA	NA	NA
Torheim et al. 2010 [55]	How to cope with the mask? Experiences of mask treatment in patients with acute chronic obstructive pulmonary disease-exacerbations	Qualitative descriptive	Norway	High-income	Adults	5	NA	COPD exacerbation	Bi-level positive airway pressure

COPD – chronic obstructive pulmonary disease, CPAP – continuous positive airway pressure, ECMO – extracorporeal membrane oxygenation, FM – facemask, ICU – intensive care unit, NA – not available, NICU – neonatal intensive care unit, NP – nasal prongs, NPO – nasopharyngeal oxygen, PACU – post-anaesthesia care unit, VLBW – very low birth weight, x̄ – mean

Between 2000–10 five studies were published [18,39,41,42,55], whereas 20 studies were published between 2011–24 [15,32,34-38,40,43-56], with 36% (n/N = 9/25) of the included articles being published in 2022, 2023, or 2024 [34,36,38,43,45,48,49,51,52]. Only two articles investigated the use of oxygen therapy for COVID-19, so the pandemic alone cannot account for the recent increase in publications. However, it may reflect an increased interest in health systems research and a patient-centred approach to health care.

In terms of study designs, 56% (n/N = 14/25) were descriptive qualitative studies [15,35-37,40,41,43,44,48-50,52,54,55]. Randomised trials were the next most common study design, accounting for 28% (n/N = 7/25) of included articles [18,38,39,42,45,46,53]. These mostly investigated patient comfort, experience, and preference while comparing different oxygen delivery methods. This information influences the patient and caregivers’ acceptability of oxygen therapy, as is detailed in the Burden section of the results, and led to the creation of the ‘medical oxygen delivery device’ component of the context section of the modified TFA.

### Theoretical framework of acceptability

**Figure 3:**
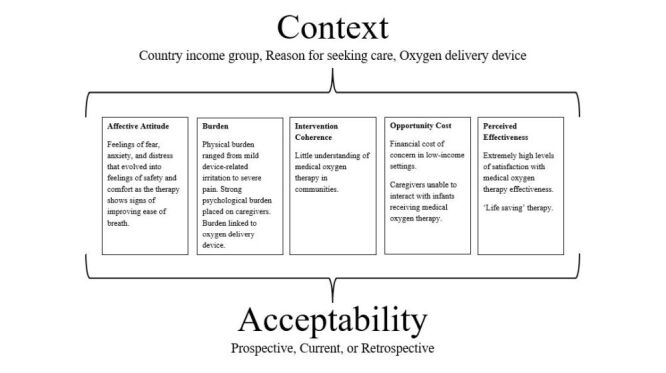
Included articles charted according to the adapted theoretical framework of acceptability.

**Figure 3** summarises the findings mapped to the adapted TFA, with the findings elaborated under each domain. Full data extraction is presented in Table S3 in the **Online Supplementary Document**. The components of ethicality (the extent to which the intervention is a good fit with an individual’s value system) and self-efficacy (the participant’s confidence that they can perform the behaviour(s) required to participate in the intervention) were removed from the original TFA as they were not reported on in any of the included papers.

### Affective attitude

Of the 25 included studies, 17 reported on the participants’ attitudes and feelings toward oxygen therapy [15,18,34-37,40-46,48,52,54,55]. Feelings of fear, distress, and anxiety were commonly reported both for the patient undergoing treatment and for caregivers on behalf of their children receiving therapy. Patients and caregivers experience fear due to the acute condition itself, but also potential real or imagined long-term effects of oxygen use [15,43,44,54]. Feelings of entrapment due to masks and tubes also increase distress and anxiety for the patient [40,48,55]. The most extreme example of these feelings is described in a study from Malawi, which reported that 83% of mothers specifically shared hearing negative opinions of oxygen therapy in the community, often using the phrase ‘oxygen kills’ [54]. These feelings influence prospective acceptability, as patients and caregivers may be reluctant to seek therapy due to prior misconceptions [15,34,43,44,54].

However, publications reported that patients and caregivers’ attitudes and feelings toward oxygen often improve once the treatment begins to work, with both patients and caregivers becoming more comfortable with the therapy as symptoms decrease [36,37,41,43,52]. Adult patients receiving treatment describe feelings of ‘safety’ and ‘comfort’ as they begin to feel the flow of oxygen [41,52], and caregivers report a similar change in perspective as they observe an improvement in their dependent’s condition [37,43]. Receiving detailed information about oxygen therapy and the equipment used from a health care professional also alleviated feelings of fear and stress [15,36,43,44]. The patient and caregiver perception of health care workers is important as trust in the care provider translates into trust of the oxygen therapy [50]. Affective attitude is thus highly malleable and dependent on the participant’s unique experience.

### Burden

Of the 25 included studies, 18 reported on the burden of undergoing oxygen therapy [18,32,35-39,41-43,46-49,51,52,54,55]. The physical burden placed on the patient included mild device-related irritation and soreness as well as breathlessness [18,36,38,39,41,47,48,51,55]. Caregivers were especially concerned with the physical burden placed upon the child, noting crying, bruising, and rashes [37,42,43,54]. Concern for the child’s physical burden was a major source of anxiety and stress for the caregivers and part of the psychological burden placed upon them [42]. In Cervantes et al. study from Canada, one mother described how she could not bear to observe the intubation process, as it felt like an assault on her infant [37]. The patients receiving oxygen therapy were also reported to endure a psychological burden, with feelings of entrapment and claustrophobia regularly reported [32,35,38,48,49]. Patients seem to report more severe psychological than physical burdens, with the helmet-continuous positive airway pressure (CPAP) therapy in particular being linked to strong feelings of entrapment [40,48]. For some, the experience is so unbearable that they would almost prefer to die than to continue enduring it [48].

The burden that oxygen therapy places upon the patient and their caregiver is an important factor in determining preference for the type of oxygen delivery method, with both groups often preferring the treatment that places a lower real or perceived burden on the patient. Ayhan et al. reported that patient satisfaction was higher for nasal cannula compared to facemasks, with the latter being more likely to be removed due to nausea and vomiting [18]. Lucchini et al. found that patients were able to tolerate the helmet CPAP with counterweights for longer than the helmet CPAP with armpit straps as it is less painful [47]. Klingenberg et al reported that the parents in their study preferred heated humidified high flow nasal cannula to nasal CPAP for their children as they perceived their child to be more comfortable and were more able to interact with them [46]. Conversely, Cirit Ekiz et al. did not find a difference in patient compliance between full face masks and oronasal masks, despite the former group being more likely to suffer from pressure ulcers, and the latter suffering more from pressure and burning sensation in the eyes [38].

Burden has a large influence on concurrent acceptability – how acceptable the therapy is whilst being received. This is especially true for the patient. If the burden becomes intolerable, they may decide to withdraw from receiving oxygen therapy [18,47].

### Intervention coherence

Of the 25 included studies, 14 investigated the extent to which participants understood oxygen therapy [15,34,36,37,39,40,43,44,48-50,52,54,55]. Universally, patients and caregivers demonstrated incomplete and misunderstandings of oxygen therapy and oxygen delivery devices. Most participants stressed the importance of receiving a comprehensive explanation of the treatment and devices and recognised the role this played in increasing their acceptance of the therapy [15,40,43,44,49,55]. On the other hand, certain caregivers in Cervantes et al.’s study expressed that the information received on potential complications from oxygen delivery was ‘a source of concern’ [37]. Nevertheless, better intervention coherence of oxygen therapy generally positively influenced acceptability. This was highlighted by participants in the Stevenson et al. study in Malawi, which suggested patient acceptance of oxygen therapy may improve with ‘better education about oxygen’ and strategies such as leaflets, posters, and interpersonal communication at health care facilities may be approaches to address this [15].

Unfortunately, acute hypoxemic events are often accompanied by impaired cognitive function in the patient, meaning improving intervention coherence before providing oxygen therapy can be difficult. Multiple studies reported their participants having an incomplete recollection of the hospitalisation process, with participants being restless and uncooperative during these events [39,48,52]. Some participants even recounted visual hallucinations, which is not uncommon due to dyspnoea [48]. These reasons, coupled with the urgency of the situation, may explain why health care workers focus on applying treatment at the expense of ensuring proper intervention coherence.

Intervention coherence plays a large part in prospective acceptability as a better understanding of oxygen therapy would help dispel some of the fears reported under affective attitude. It also influences concurrent acceptability as both patients and caregivers are more likely to tolerate the burden of the treatment if they understand its necessity [40,44,49].

### Opportunity cost

Of the 25 included studies, 11 reported on the opportunity cost of oxygen therapy [15,34,36,37,41,42,44,46,48,50,52]. The financial burden of oxygen was only reported in low- and middle-income countries (LMICs), with 78% of patients and 87% of caregivers in Adeoti et al. study in Nigeria expressing that ‘oxygen was too expensive and a barrier to its procurement and administration’ [34]. Participants in the Stevenson et al. study in Malawi were also reluctant to be treated with oxygen due to ‘concerns about the cost of oxygen’ [15]. Multiple studies also reported that patients were unable to perform routine daily activities such as eating, talking, and going to the bathroom while receiving oxygen treatment [36,41,47,52].

The inability to hold and comfort their child was a major source of stress for caregivers [37,42,44,50]. Foster et al. found that this separation from the baby was the highest reported stressor for parents, even more so given their child is sick (*e.g.* ‘baby seemed in pain’) [42]. The lack of physical contact between parent and child is included under opportunity cost due to the importance of neonatal skin-to-skin contact, or kangaroo care, in improving maternal-infant bonding, reducing stress levels, and improving the infant’s immune system and brain development [57]. Opportunity cost may affect prospective acceptance as patients or caregivers could be unable or delay seeking treatment if the cost is too high.

### Perceived effectiveness

Of the 25 included studies, 16 reported on the perceived effectiveness of oxygen therapy [34-37,40-45,48-50,53-55]. Oxygen was almost universally perceived as effective at reducing symptoms of hypoxemia by patients and caregivers alike, with five studies independently reporting their participants using some variation of the term ‘life saving’ while describing the treatment [35,37,44,48,55]. Importantly, this high level of perceived effectiveness often influenced affective attitude, with participants expressing more positive feelings about oxygen therapy once they experienced or saw the treatment performing as intended. This is especially the case in Gebre et al. study in Nigeria, where caregivers were initially ‘anxious and doubtful’ about the effectiveness of bubble CPAP treatment until a few hours later when they became ‘happy, relaxed, and satisfied’ when they observed that symptoms were slowly disappearing [43].

Of all the included components of the acceptability framework, perceived effectiveness seems to be the most universally consistent between publications and the most positive. Perceived effectiveness plays a large part in retrospective acceptability as most participants are left convinced that oxygen therapy is an acceptable treatment after experiencing its therapeutic effects.

## DISCUSSION

We aimed to describe the current evidence on patient and caregiver perceptions of oxygen treatment in facility-based settings for acute medical conditions. By applying an adapted version of the TFA to the included studies, we explored participant perspectives concerning affective attitude, burden, intervention coherence, opportunity cost, and perceived effectiveness. It was clear that affective attitude is strongly influenced by perceived effectiveness – which was almost universally positive – and that patients and their caregivers suffer different types of burdens. Additionally, we found that intervention coherence in all represented communities was low and that opportunity costs were highest in LMICs and for caregivers of neonates. The lack of publications from South and East Asia and Latin America is an important finding, and highlights areas for further research, given the importance of context for some of the TFA domains.

Applying the TFA identified components of oxygen therapy that were more universally experienced. Perceived effectiveness was the most universal component of acceptability, with all included studies, regardless of country and patient group, reporting that their participants felt like the treatment was beneficial and even life-saving. Patient and caregiver experiences while receiving oxygen therapy were also similar across settings, with a comparable burden irrespective of the environment, and patients and caregivers both shouldering forms of psychological burden. Similarly, intervention coherence was low regardless of the setting and none of the included studies reported participants having a thorough understanding of oxygen therapy. We also noted that different components of acceptability are inter-linked and influence each other. When considering the temporal perspectives of acceptability, prospective acceptability was shaped by affective attitude, intervention coherence, and opportunity cost, while concurrent acceptability was largely defined by burden and intervention coherence, and retrospective acceptability was strongly reliant on perceived effectiveness.

Differences between geographical regions were found in affective attitude. Only studies from low-income countries – namely Malawi and Ethiopia – reported high levels of misinformation about oxygen therapy in the community [15,43,44,54]. All three studies situated in Malawi reported on participants believing that oxygen therapy or the oxygen delivery device is associated with death [15,44,54]. This can be explained as a rational fear, given the higher prevalence of diseases requiring acute oxygen treatment, such as pneumonia and severe malaria in LMICs [5,6,58], and often late presentation to care, which results in high inpatient mortality rates [1]. This can lead to a confirmation bias, *i.e.* those who are sickest are given oxygen, but are also most likely to die. These poor health outcomes while receiving oxygen treatment can then spread through the community and delay care-seeking, driving a negative feedback loop. This is supported by a subsequently published paper which found that in Nigeria, non-acceptance of oxygen therapy for sick children was associated with misconceptions about the treatment [59]. However, this paper also reported that these negative perceptions can be modified when positive experiences with oxygen are normalised, presenting opportunities to intervene.

Educational campaigns have demonstrated an ability to increase community understanding for a range of conditions and public health issues and their associated medical interventions [60,61]. Increasing knowledge can potentially lead to increased uptake of medical interventions and decreased anxiety surrounding the treatment [61-65]. Public health initiatives to change perceptions of acute medical oxygen should be inspired by previous successful interventions in the same settings. They should use tools such as awareness campaigns, community-based education, and positive stories of medical oxygen (*e.g.* television dramas) [59]. While misinformation was specifically an issue found in papers from low-income settings, the COVID-19 pandemic and accompanying ‘infodemic’ – defined by WHO as an overabundance of information, with mixed reliability that occurs during an epidemic – has shown that no community is safe from ‘fake news’ and conspiracy [66-68]. However, settings with lower health literacy, poorer health infrastructure, and underlying distrust in government agencies may be more susceptible to its negative effects [67]. Therefore, active strategies to combat misinformation from arising should be undertaken, especially considering the dominant role of oxygen as a treatment during pandemics, disasters and conflicts. Our finding of misconceptions around oxygen supports the WHO’s ‘Access to Medical Oxygen’ resolution asking member states to ‘raise public awareness, as appropriate, about the life-saving role of medical oxygen as a treatment for many conditions’ [10].

The opportunity cost of oxygen therapy was also experienced differently across regions, with direct financial cost only being reported in LMICs – Malawi and Nigeria [15,34]. Previous studies investigating barriers to implementing oxygen therapy in low-income settings have also identified affordability and cost-effectiveness of oxygen as important facilitators [69,70]. To improve the uptake of medical oxygen in settings where health care is not free and the costs associated with seeking care are high, schemes that subsidise or remove user fees need to be considered. While these are not easy solutions, equitable access to care is a global health priority, and therefore, oxygen should be part of essential service packages and universal health care.

Throughout the included studies, adult patients and adult caregivers reported similar experiences across most domains, except burden. Understandably, caregivers are not subjected to the physical discomfort of treatment (which was specific to the oxygen delivery device) or the associated feelings of entrapment and claustrophobia. Instead, caregivers reported elevated levels of stress and anxiety, particularly during the initiation of the intervention and prior to the patient experiencing the positive effects of oxygen treatment. These feelings were not unique to parents of children undergoing oxygen therapy but are shared between caregivers of hospitalised adults [71-74]. These findings were consistent across country income group classifications. A previous study demonstrated a correlation between the patient’s age and their parents’ stress levels during hospitalisation, with parents of younger children found to be more stressed [71]. This matches our finding that parents of infants face larger opportunity costs. Introducing support mechanisms for caregivers of hospitalised patients is a viable solution and may even improve child hospital outcomes [74]. Simply improving caregiver-provider communication can have a strong impact on the former’s mental health status and satisfaction with care in neonatal intensive care units [73]. Again, this is consistent with results from this review that increasing intervention coherence in caregivers increased acceptance of the treatment and reduced stress levels [15,40,43,44,49].

To the best of our knowledge, this is the first scoping review to study patient and caregiver experience of oxygen therapy applying the TFA. However, we had three limitations. First, despite including Spanish and French language papers, we only conducted the searches in global English language databases. The search only returned a single non-English paper, and it is possible that we missed papers, especially from Latin America and South and East Asia, where no papers were included. We ran a post-hoc search in Google Scholar to check, using translated terms for ‘oxygen,’ ‘patient/caregiver,’ and ‘perspective’ in Spanish and Chinese, and no additional studies were identified. We, therefore, think this reflects an important knowledge gap in the available literature rather than a limitation of this paper. Second, other sources of data from grey literature and industry (*e.g.* for product development and piloting) were not included. These may have provided different perspectives, but were outside our scope. Finally, a snowball reference search was not performed, so some studies may have been missed.

## CONCLUSIONS

Overall, we found oxygen to be a universally accepted treatment by patient and caregivers, but not without hesitations and burdens, that varied across patient groups, geographical settings and oxygen delivery devices. Intervention coherence was singled out as a key policy area for improvement to help increase acceptability of oxygen therapy, and future interventions could focus on initiatives to increase access to reliable information about oxygen, and improving communication during clinical care. The high cost of oxygen was also a barrier, and one that was inequitably experienced, and we therefore strongly support the need for universal health coverage that includes oxygen therapy for all patients in need.

## Additional material


Online Supplementary Document
