# Supplementary material for: Patient and caregiver perceptions of oxygen therapy in facility-based settings for acute hypoxemic conditions: a scoping review
Source: J Glob Health. 2025 Apr 25;15:04084. doi: 10.7189/jogh.15.04084 (PMC12022931; doi:10.7189/jogh.15.04084)
Supplement: Online Supplementary Document [file jogh-15-04084-s001.pdf]

Karolinska Institutet

Supplement to: Britto M, Bakare AA, Graham H, King C. Patient and caregiver perceptions of oxygen therapy in facility-based settings for acute hypoxemic conditions: a scoping review. J Glob Health. 2025;15:04084.

Figure S1: Search strategies

1. Medline

Interface: Ovid MEDLINE(R) and Epub Ahead of Print, In-Process & Other Non-Indexed Citations and Daily<br><br>Date of Search: 27 December 2022<br><br>Number of hits: 4,369<br><br>Comment: In Ovid, two or more words are automatically searched as phrases; i.e. no quotation marks are needed	Field labels<br><br>• exp/ = exploded MeSH term<br>• / = non exploded MeSH term<br>• .ti,ab,kf. = title, abstract and author keywords<br>• adjx = within x words, regardless of order<br>• * = truncation of word for alternate endings

Database(s): **Ovid MEDLINE(R) ALL** 1946 to December 23, 2022
Search Strategy:

#	Searches	Results
1	Continuous Positive Airway Pressure/	8942
2	exp Oxygen Inhalation Therapy/	28177
3	Oxygen Saturation/	880
4	exp Oximetry/	16528
5	Oxygen/	174938
6	(BPAP* or B-PAP* or BCPAP* or B-CPAP* or BiPAP* or bi-PAP* or CPAP* or C-PAP* or nCPAP* or n-PAP* or nasal cannula* or nasal catheter* or nasal prong* or nasal tube* or oximet*).ti,ab,kf.	32256
7	((automatic* or bilevel* or bi-level* or biphasic* or bi-phasic* or biphasic* or bubble* or continuou* or constant* or ventilat*) adj3 airway pressure*).ti,ab,kf.	13137
8	((oxygen* or O2) adj3 (high-flow* or highflow* or low-flow* or lowflow* or mask* or monitor* or saturat* or supplement* or therap* or treat*)).ti,ab,kf.	72327
9	or/1-8	268749
10	Caregivers/	47396
11	Family/	83570
12	exp Parents/	136384
13	Patients/	22682

14	Spouses/	11407
15	or/10-14	281026
16	(caregiver* or care-giver* or carer* or caretaker* or communit* or father* or famil* or husband* or mother* or maternal* or "next-of-kin" or parent* or partner* or paternal* or patient* or person* or population* or significant other* or spouse* or wife or user*).ti,ab,kf.	11517748
17	15 or 16	11556522
18	Attitude/	52644
19	Informed Consent/	38432
20	exp "Patient Acceptance of Health Care"/	171221
21	Perception/	42233
22	Therapeutic Misconception/	175
23	"Treatment Adherence and Compliance"/	1003
24	Treatment Refusal/	12100
25	or/18-24	306794
26	psychology.fs.	1164054
27	(accept* or adher* or attitud* or barrier* or complian* or consent* or concept* or experienc* or misconcep* or mis-concep* or nonaccept* or nonadheren* or noncomplian* or opinion or participat* or percept* or perceiv* or perspect* or refus* or uptake* or up-take* or view*).ti,ab,kf.	4986265
28	25 or 27	5089768
29	((caregiver* or care-giver* or carer* or caretaker* or communit* or father* or famil* or husband* or mother* or maternal* or "next-of-kin" or parent* or partner* or paternal* or patient* or person* or population* or significant other* or spouse* or wife or user*) adj3 (accept* or adher* or attitud* or barrier* or complian* or consent* or concept* or experienc* or misconcep* or mis-concep* or nonaccept* or nonadheren* or noncomplian* or opinion? or participat* or percept* or perceiv* or perspect* or refus* or uptake* or up-take* or view*)).ti,ab,kf.	582315
30	15 and 26	134996
31	15 and 28	127421
32	17 and 25	200798
33	or/29-32	833853
34	9 and 33	5480
35	limit 34 to (clinical conference or consensus development conference or consensus development conference, nih)	7
36	34 not 35	5473

37	limit 36 to (english or french or spanish)	5145
38	limit 37 to yr="2000 -Current"	4369

2. Embase

Interface: embase.com<br><br>Date of Search: 27 December 2022<br><br>Number of hits: 7,733<br><br>Comment: Emtree is the controlled vocabulary in Embase	Field labels<br><br>• /exp = exploded Emtree term<br>• /de = non exploded Emtree term<br>• ti,ab,kw = title, abstract and author keywords<br>• NEAR/x = within x words, regardless of order<br>• * = truncation of word for alternate endings

No.	Query	Results
#1	'continuous positive airway pressure'/exp	7828
#2	'hyperbaric oxygen therapy'/de	20491
#3	'nocturnal oxygen therapy'/de	63
#4	'nasal cannula therapy'/exp	4994
#5	'nasal cannula'/exp	8082
#6	'oximetry'/exp	34466
#7	'oxygen'/de	253696
#8	'oxygen mask'/exp	3283
#9	'oxygen saturation'/de	72842
#10	'oxygen therapy'/de	42452
#11	bpap*:ti,ab,kw OR 'b-pap*':ti,ab,kw OR bcpap*:ti,ab,kw OR 'b-cpap*':ti,ab,kw OR bipap*:ti,ab,kw OR 'bi-pap*':ti,ab,kw OR cpap*:ti,ab,kw OR 'c-pap*':ti,ab,kw OR ncpap*:ti,ab,kw OR 'n-pap*':ti,ab,kw OR 'nasal catheter*':ti,ab,kw OR 'nasal cannula*':ti,ab,kw OR 'nasal prong*':ti,ab,kw OR 'nasal tube*':ti,ab,kw OR oximet*:ti,ab,kw	54149
#12	((automatic* OR bilevel* OR 'bi-level*' OR biphasic* OR 'bi-phasic*' OR biphasic* OR bubble* OR continuou* OR constant* OR ventilat*) NEAR/3 'airway pressure*'):ti,ab,kw	19575
#13	((oxygen* OR o2) NEAR/3 ('high-flow*' OR highflow* OR 'low-flow*' OR lowflow* OR mask* OR monitor* OR saturat* OR supplement* OR therap* OR treat*)):ti,ab,kw	103038
#14	#1 OR #2 OR #3 OR #4 OR #5 OR #6 OR #7 OR #8 OR #9 OR #10 OR #11 OR #12 OR #13	445093
#15	'caregiver'/de	105523
#16	'community'/de	85522
#17	'family'/de	105540
#18	'patient'/de	1461260

Karolinska Institutet

#19	'spouse'/exp	23569
#20	#15 OR #16 OR #17 OR #18 OR #19	1725715
#21	caregiver*:ti,ab,kw OR 'care giver*':ti,ab,kw OR carer*:ti,ab,kw OR caretaker*:ti,ab,kw OR communit*:ti,ab,kw OR father*:ti,ab,kw OR famil*:ti,ab,kw OR husband*:ti,ab,kw OR mother*:ti,ab,kw OR maternal*:ti,ab,kw OR 'next-of-kin':ti,ab,kw OR parent*:ti,ab,kw OR partner*:ti,ab,kw OR paternal*:ti,ab,kw OR patient*:ti,ab,kw OR person*:ti,ab,kw OR population*:ti,ab,kw OR 'significant other*' OR spouse*:ti,ab,kw OR wife:ti,ab,kw OR user*:ti,ab,kw	15830953
#22	#20 OR #21	15875310
#23	'attitude'/de	74879
#24	'informed consent'/de	127304
#25	'patient compliance'/exp	182863
#26	'patient attitude'/de	76010
#27	'patient participation'/de	33790
#28	'perception'/de	157780
#29	'therapeutic misconception'/de	1113
#30	'treatment refusal'/de	22114
#31	#23 OR #24 OR #25 OR #26 OR #27 OR #28 OR #29 OR #30	639227
#32	accept*:ti,ab,kw OR adher*:ti,ab,kw OR attitud*:ti,ab,kw OR barrier*:ti,ab,kw OR complian*:ti,ab,kw OR consent*:ti,ab,kw OR concept*:ti,ab,kw OR experienc*:ti,ab,kw OR misconcep*:ti,ab,kw OR 'mis-concep*':ti,ab,kw OR nonaccept*:ti,ab,kw OR nonadheren*:ti,ab,kw OR noncomplian*:ti,ab,kw OR opinion$:ti,ab,kw OR participat*:ti,ab,kw OR percept*:ti,ab,kw OR perceiv*:ti,ab,kw OR perspect*:ti,ab,kw OR refus*:ti,ab,kw OR uptake*:ti,ab,kw OR 'up-take*':ti,ab,kw OR view*:ti,ab,kw	6605599
#33	#31 OR #32	6792076
#34	((caregiver* OR 'care-giver*' OR carer* OR caretaker* OR 'care-taker*' OR communit* OR father* OR famil* OR husband* OR mother* OR maternal* OR 'next-of-kin' OR parent* OR partner* OR paternal* OR patient* OR person* OR population* OR 'significant other*' OR spouse* OR wife OR user*) NEAR/3 (accept* OR adher* OR attitud* OR barrier* OR complian* OR consent* OR concept* OR experienc* OR misconcep* OR 'mis-concep*' OR nonaccept* OR nonadheren* OR noncomplian* OR opinion$ OR participat* OR percept* OR perceiv* OR perspect* OR refus* OR uptake* OR 'up-take*' OR view*)):ti,ab,kw	872963
#35	#20 AND #33	555248
#36	#22 AND #31	421652
#37	#34 OR #35 OR #36	1524001
#38	#14 AND #37	18963
#39	#38 AND ('Conference Abstract'/it OR 'Conference Paper'/it OR 'Conference Review'/it)	9948
#40	#38 NOT #39	9015
#41	#38 NOT #39 AND ([english]/lim OR [french]/lim OR [spanish]/lim)	8504
#42	#38 NOT #39 AND ([english]/lim OR [french]/lim OR [spanish]/lim) AND [2000-2023]/py	7733

3. Web of Science Core Collection

Interface: Clarivate Analytics<br><br>Editions = A&HCI , ESCI , SCI-EXPANDED , SSCI<br><br>Date of Search: 27 December 2022<br><br>Number of hits: 2,277	Field labels<br><br>• TS/Topic = title, abstract, author keywords and Keywords Plus<br>• NEAR/x = within x words, regardless of order<br>• * = truncation of word for alternate endings<br><br>Note: the *Exact search*-function was used for all the searches

#	Search Query	Results
1	TS=((oxygen* OR o2) NEAR/2 ("high-flow*" OR highflow* OR "low-flow*" OR lowflow* OR mask* OR monitor* OR saturat* OR supplement* OR therap* OR treat*))	73582
2	TS=((automatic* OR bilevel* OR "bi-level*" OR biphasic* OR "bi-phasic*" OR biphasic* OR bubble* OR continuou* OR constant* OR ventilat*) NEAR/2 "airway pressure*")	12938
3	TS=(bpap* OR "b-pap*" OR bcpap* OR "b-cpap*" OR bipap* OR "bi-pap*" OR cpap* OR "c-pap*" OR ncpap* OR "n-pap*" OR "nasal catheter*" OR "nasal cannula*" OR "nasal prong*" OR "nasal tube*" OR oximet*)	36988
4	#1 OR #2 OR #3	104974
5	TS=((caregiver* OR "care giver*" OR carer* OR caretaker* OR communit* OR father* OR famil* OR husband* OR mother* OR maternal* OR "next-of-kin" OR parent* OR partner* OR paternal* OR patient* OR person* OR population* OR "significant other*" OR spouse* OR wife OR user*) NEAR/2 (accept* OR adher* OR attitud* OR barrier* OR complian* OR consent* OR concept* OR experienc* OR misconcep* OR "mis-concep*" OR nonaccept* OR nonadheren* OR noncomplian* OR opinion$ OR participat* OR percept* OR perceiv* OR perspect* OR refus* OR uptake* OR "up-take*" OR view*))	664656
6	#5 AND #4	3282
7	#5 AND #4 and Meeting Abstract or Proceeding Paper (Document Types)	229
8	#6 NOT #7	3053
9	#8 AND (LA==("ENGLISH" OR "FRENCH" OR "SPANISH"))	2958
10	#9 Timespan: 2000-01-01 to 2022-12-31	2677

Table S1: Quality appraisal

a) Qualitative studies

Reference	Clear statement of Aims & Research	Is a qualitative methodology appropriate?	Was the research design appropriate to address the aims of the research?	Was the recruitment strategy appropriate to the aims of the research?	Was the data collected in a way that addressed the research issue?	Has the relationship between researcher and participants been adequately considered?	Have ethical issues been taken into consideration?	Was the data analysis sufficiently rigorous?	Is there a clear statement of findings?	How valuable is the research?
Adeoti (2022)										
Beckert (2020)										
Bitterman (2016)										
Brugnolli (2023)										
Cervantes (2011)										
Dimech (2012)										
Eastwood (2009)										
Gebre (2022)										
Gondwe (2017)										
Lucchini (2023)										
McCormick (2022)										
Peeler (2015)										
Sandau (2022)										
Sessions (2020)										
Stevenson (2015)										
Torheim (2010)										

b) Quantitative studies

Reference	Did the study address a clearly focused research question?	Was the assignment of participants to interventions randomised?	Were all participants who entered the study accounted for at its conclusion?	Were the participants and investigators blinded?	Were the study groups similar at the start of the randomised controlled trial?	Apart from the experimental intervention, did each study group receive the same level of care (that is, were they treated equally)?	Were the effects of intervention reported comprehensively?	Was the precision of the estimate of the intervention or treatment effect reported?	Do the benefits of the experimental intervention outweigh the harms and costs?	Can the results be applied to your local population/in your context?	Would the experimental intervention provide greater value to the people in your care than any of the existing interventions?
Ayhan (2009)											
Betensley (2008)											
Cirit Ekiz (2022)											
Constantin (2009)											
Foster (2008)											
Hansen (2018)											
Klingenberg (2014)											
Lucchini (2019)											
Peterson (2023)											
Sandau (2023)											

Table S3 – Data extraction for the Theoretical Framework of Acceptability

Author (date)	Affective attitude	Burden	Intervention Coherence	Opportunity Cost	Perceived Effectiveness
Adeoti (2022)	The majority [of participants] would accept the		21.1% of patients and 19.5% of caregivers believed it is	A major proportion of patients and caregivers (patients vs.	The majority of the participants (patients vs.

	administration of oxygen, if the need arises (Patients vs Caregivers, 84.7% vs 81.4%)		solely for terminally ill patients; More than 50% of patients and caregivers thought oxygen can cause adverse effects and fire outbreaks.	caregivers, 78.2% vs. 87.2%) felt that oxygen was too expensive and a barrier to its procurement and administration.	caregivers 84.7% vs. 81.1% P = 0.511) believed oxygen therapy to be beneficial and safe for use (patients vs. caregivers 79.4% vs. 78.6%; P = 0.895)
Ayhan (2009)	Average satisfaction scores for patients in the nasal cannulae group were statistically significantly higher than those in the mask group	Oxygen masks were removed by the majority of patients (n = 19/40). The primary reason for removal was a feeling of distress (68·3%); nasal cannulae were removed by only two patients, caused by nose pain.			
Beckert (2020)	Describing NIV as a safety mechanism and providing rationalisation about the mask	Participants reported that they disliked their experience with the mask. They noted that it was often too tight and made them claustrophic with feelings of suffocation and loss of control, which made it difficult for them to relax; Participants described how they learnt to synchronise their breathing with the NIV, which made the experience more bearable. They reported that it was difficult to maintain the synchronisation over a long period of time			Participants shared many stories of NIV as a 'life saver'.
Bitterman (2016)		Psychological manifestations reported include isolation, claustrophobia and anxiety, insufficiency of control or autonomy, boredom, and lack of personal space and privacy;			

		The hyperbaric chamber is a noisy environment that disrupts communication between people inside the chamber and the outside with control panels and staff, leading to anxiety and restlessness			
Brugnolli (2023)	they report feelings of helplessness, fear, uncertainty and feeling close to the limit; fears of suffocation given by 'wearing something that wraps around your whole head' or 'being enclosed in a bag' (P 18, 5); an initial frightening impact from the feeling of not breathing and gradually the perception of benefit and relief given by their use.	Deafening noise and discomfort, dryness of the air and pressure from the suspenders and sheathing	All respondents appreciated the 'humanity' of HCPs and perceived the encouragement they received and the 'reassuring presence' as decisive in overcoming discomfort and difficulties. 'He explained what I was up against, I get emotional thinking about it, he held my hands and very calmly explained the seriousness of it, but with great humanity. He really gave me courage' (P 4).	Participants described helmet as a barrier between them and reality, using the terms like 'flask', 'diving suit' and one of them states: 'you are like a fish inside an aquarium, you see others, but you can't scream and communicate' (P 5).	Participants also gradually expressed the perceived and seen benefit of the helmet and pronation, mostly related to the 'saturation improvement': 'With the helmet I could see from the monitor that my saturation was good. When they took my helmet off to eat, … it was drama. With the slightest movement, even simply swallowing, I would see the saturation drop 90–89… and I would say: -oh my God put my helmet back on before it drops any more' (P 20).
Cervantes (2011)	Mothers described the methods of delivering oxygen as initially overwhelming and distressing. Nonetheless, with time they learned about it and became increasingly comfortable with the equipment.	Mothers spoke of the discomfort they imagined their infants felt with each type of oxygen delivery method. Mothers perceived that the intubation process was the most painful for their infants and suggested that the presence of blood after intubation was evidence of their infants' pain. One	Information received in the NICU from staff and other parents about potential complications from oxygen delivery was a source of concern. However, most mothers explained that overall these potential negative consequences were offset by the positive benefits of oxygen therapy.	This study's participants described that their inability to see their infants' face or hear their voice, and not being able to hold their infants as particularly distressing aspects of various methods of oxygen delivery	All the mothers perceived that that oxygen therapy had kept their infants alive

		mother described the intubation process as an “assault” on her infant that she could not watch.			
Cirit Ekiz (2022)		During the treatment, [...] five patients [from the full-face mask group were excluded from the study] due to claustrophobia; The complaints of burning sensation and pressure in the eyes were higher in the full face mask group (p= 0.025)			
Constantin (2009)		Sophrology allowed a significant reduction in digital visual scale values in terms of reduction of discomfort (−60%), respiratory discomfort (−76%) and reduction of pain (−40%) (p < 0.001)	Restlessness and uncooperativeness resulting from pain, anxiety, and discomfort		
Dimech (2012)	Throughout the interviews feelings of entrapment were described.		Some of the patients interviewed described confusing messages that they received from health care professionals during their treatment with helmet CPAP. Patient B found that in his experience some of the nursing staff did not explain the therapy and some seemed to lack knowledge in the management of the helmet CPAP; some patients explained that they		all patients interviewed comment upon the environment within the helmet CPAP and its ability to help them breathe.

			commenced or continued treatment as they felt it was appropriate because of advice received from the health care professionals.		
Eastwood (2009)	Some [patients] felt reassured to know that they were receiving oxygen and mentioned that the flow of oxygen through the FM gave them a sense of comfort.	[patients] described device-related soreness, irritation and poor fit as contributing factors to feelings of discomfort.		Talking, eating and drinking were common activities that occurred while receiving oxygen therapy. Patients commented that the devices at times restricted these activities.	Patients considered all three low-flow oxygen therapy devices safe and effective.
Foster (2008)	Parents with babies receiving CPAP rated their satisfaction with the baby's treatment statistically significantly higher than the headbox group mean rating	Stress was perceived as at least moderate by a majority of parents; 'Baby looks in pain' and 'baby looking sad' were the most potent individual stressors for our parents within the scale 'infant behaviour and appearance'.		The highest reported stress occurred for 'separation from the baby'.	Parents of infants receiving CPAP in our study were 'extremely satisfied' with their infant's treatment compared to 'very satisfied' for the parents of infants receiving headbox oxygen.
Gebre (2022)	Initially, caregivers were anxious and doubtful about the positive outcome of bCPAP treatment, until they began to observe improvement of breathing difficulty of their children; caregivers perceived that bCPAP might be harmful to their children	[Caregivers] were concerned about the child's crying with flushed face, dry nose, and the noise of oxygen concentrator; children felt mild discomfort at the beginning of fixing the nasal prong into their nostrils	Later the clinicians explained to them how bCPAP oxygen therapy works, which made caregivers happy and relieved their anxiety.		After few hours of bCPAP treatment, all caregivers were happy, relaxed, and satisfied when they observed all signs and symptoms (e.g. fever, cough, vomiting, sneezing) were slowly disappearing and the condition of their children improved (easier breathing, sleeping, spontaneous eye opening, smiling, eating, playing), which was beyond their expectations.
Gondwe (2017)	Participants had varying reactions to bCPAP but most		Participants knew that bCPAP was treatment to provide extra	the caregivers reported that the tubing on bCPAP	when caregivers' infants were on bCPAP, caregivers were

	reported fear when they saw their infants on bCPAP. They feared that their children may die or be hurt by the machine. Some participants expressed feelings such as broken heartedness, worry, disappointment, and anxiety; Most participants had prior misconceptions that oxygen delivering devices (nasal prong and bCPAP) kill.		oxygen to infants who were in severe respiratory distress; Many participants acknowledged that the information helped them to understand their infant's condition and bCPAP treatment. Among the participants who were told about bCPAP before it was commenced, a few participants explained that the information helped them to accept the treatment	machines prevented contact between them and their children	satisfied and concluded that it was good. Some participants described it as "lifesaving".
Hansen (2018)	Patients' acceptance of automated oxygen delivery in our study was very high				Eight (62%) expressed very high confidence in getting the right amount of oxygen, four (31%) expressed quite a bit confidence, and one (8%) did not know. None expressed little or no confidence.
Klingenberg (2014)	The parents preferred HHHFNC as respiratory support for their infants	we found no important differences in patient comfort with HHHFNC versus NCPAP		[parents] perceived it easier to interact with their child when they were on HHHFNC	
Lucchini (2019)		patients might benefit from the use of counter-weights system thanks to better tolerance and a reduction of the pain experienced during the CPAP			
Lucchini (2024)	Common expressions were feelings of trapping, uncertainty and mental confusion, helplessness and difficult communication with	Feelings of entrapment were well-described; some participants associated entrapment with breathing difficulties. Some participants	Patients described memories of both real and surreal episodes regarding time spent with the helmet in ICU. The hallucinations were described	Separation from their loved ones; The difficulties experienced by patients in communicating with nurses	All patients expressed awareness of the importance of helmet-CPAP to improve their oxygenation and its ability to help them breathe:

	others, as well as fear of dying.	described the experience inside the helmet as a nightmare, declaring that they almost preferred to die [than] having to put the helmet back on.	as unexpected and realistic experiences.	and physicians were well remembered by participants	"it had positive effects on my health (participant nr.13, male)"; "it saved my life (interview nr.16)".
McCormick (2022)		The fit around the nose was specifically called out by multiple patients, along with the mask’s tightness, restriction, and pressure on the face; The psychological impact of feeling confined and claustrophobic with the mask on their face was reported by many patients.	Patients whose provider explained what to expect with the NIV mask, the benefits of NIV, and alternatives to NIV, felt included, informed, and ready for treatment; they were more likely to “put up” with the discomfort caused by the mask.		Some patients reported that NIV helped them breathe better immediately, taking little time for the treatment to alleviate their respiratory distress; most patients realized that the treatment was needed and helped them
Peeler (2015)			Mothers described their experience of having a child in a headbox as frustrating, anxiety ridden or fearful as they did not fully understand what was happening to their child, the treatment nor illness.	The mothers stated that they were less involved with their child when he/she was receiving headbox oxygen therapy, which was described as a barrier that made them feel isolated.	Participants were unified in their perception that high-flow nasal prong oxygen therapy provided a more positive method of treating the children [than headbox oxygen].
Peterson (2023)		The most frequently reported symptoms were thirst (86, 75.4%), anxiety (68, 59.6%), tiredness (62, 54.3%), and restlessness (61, 53.5%). Most participants endorcing the symptoms of being scared and short of breath rated as severly intense (56% and 54%, respectively) and severely distressful (55% and 60%, respectively).			

Sandau (2022)	Although the robot provided a feeling of safety, it also introduced fear and a feeling of lack of safety when the therapeutic alterations were taking some time to reach the defined SpO2 range.	The patients described how they felt restricted by the nasal cannula; the patients described how the length of the robot's oxygen tube and wires constituted both a physical and a mental barrier for their activities as it made them dependent on nursing assistance to transfer to a chair, to go to the toilet, or to walk; Our findings showed that patients were willing to compromise their own safety by switching off the AOA so it would not be a "noisy burden."	The patients referred to the first few days of their hospitalization as unclear and "blurry," and did not remember that they had received oxygen treatment through a robot.	When the patients disconnected themselves from the robot (by taking off the finger clip) to eat, go to the toilet, or get dressed, an alarm would start after five minutes. The alarm would also go off if SpO2 dropped below a given range, like after a toilet visit, and then its intensity would increase with time until SpO2 was back within range. To avoid this scenario, the patients adjusted their activities of daily living by e.g. rushing through their normal routines in the toilet.	
Sandau (2023)					The choice of "Air hunger" as the most accurate descriptor was reduced during the intervention from 26 to 6 patient choices in the intervention group as opposed to a reduction from 28 to 20 patient choices in the control group.
Sessions (2020)	Almost all information participants received from the community was negative. Eighty-three per cent of mothers (45/54) explicitly, and without prompting, mentioned hearing negative opinions of supplemental oxygen therapy in the community, many using the	A majority of the mothers had no concerns about treatment. Some mothers were concerned about bruising, rashes or nasal injury.	knowledge of oxygen, and also bCPAP, was low within the participants' communities; The knowledge about bCPAP was significantly lower; most reported they had never heard of bCPAP before being educated by the study staff at the hospital. However, those who had heard of bCPAP		After treatment, the mothers were supportive of their care regardless of her child's health outcome and perceived multiple benefits of both treatments.; The most commonly discussed benefit was that the machines help children breathe.

	phrasing that ‘oxygen kills’; community beliefs led mothers to feel anxious and fearful prior to treatment initiation. Additional sources of fear included lack of familiarity with the machine		reported similar negative concern that it can worsen the child’s condition or kill the child.		
Stevenson (2015)	A recurrent theme was fear of oxygen, often due to a perceived association between death and recent oxygen use; reluctance to be treated with oxygen due to fear of oxygen. Participants frequently perceived it to be harmful, as many had witnessed or heard of a poor outcome following oxygen use		Many participants felt they did not know enough about oxygen and the equipment used to deliver it to make informed choices about it; Participants suggested that uptake may improve with better education about oxygen and suggested strategies such as leaflets, posters and interpersonal communication at healthcare facilities.	Other reasons for reluctance to be treated with oxygen included concerns about cost of oxygen	
Torheim (2010)	The mask treatment did at times intensify the anxiety. Some patients felt trapped as a result of the feeling of not being able to breathe; Tightening the mask as soon as it was put on made some patients afraid, as did not being able to find the bell.	Not knowing how to remove the mask and having it on for a long time without a break caused anxiety; The mask also caused some physical discomforts which could be quite painful. Pressure sores on the nose, forehead and cheeks were described	All patients interviewed stated that prior knowledge of the mask would have been advantageous.		Some were convinced that the mask ‘saved their lives’.

Table S3 – Data extraction for the Theoretical Framework of Acceptability

Author (date)	Affective attitude	Burden	Intervention Coherence	Opportunity Cost	Perceived Effectiveness
Adeoti (2022)	The majority [of participants] would accept the administration of oxygen, if		21.1% of patients and 19.5% of caregivers believed it is solely for terminally ill	A major proportion of patients and caregivers (patients vs. caregivers, 78.2% vs. 87.2%)	The majority of the participants (patients vs. caregivers 84.7% vs.

	the need arises (Patients vs Caregivers, 84.7% vs 81.4%)		patients; More than 50% of patients and caregivers thought oxygen can cause adverse effects and fire outbreaks.	felt that oxygen was too expensive and a barrier to its procurement and administration.	81.1% P = 0.511) believed oxygen therapy to be beneficial and safe for use (patients vs. caregivers 79.4% vs. 78.6%; P = 0.895)
Ayhan (2009)	Average satisfaction scores for patients in the nasal cannulae group were statistically significantly higher than those in the mask group	Oxygen masks were removed by the majority of patients (n = 19/40). The primary reason for removal was a feeling of distress (68·3%); nasal cannulae were removed by only two patients, caused by nose pain.			
Beckert (2020)	Describing NIV as a safety mechanism and providing rationalisation about the mask	Participants reported that they disliked their experience with the mask. They noted that it was often too tight and made them claustrophic with feelings of suffocation and loss of control, which made it difficult for them to relax; Participants described how they learnt to synchronise their breathing with the NIV, which made the experience more bearable. They reported that it was difficult to maintain the synchronisation over a long period of time			Participants shared many stories of NIV as a ‘life saver’.
Bitterman (2016)		Psychological manifestations reported include isolation, claustrophobia and anxiety, insufficiency of control or autonomy, boredom, and lack of personal space and privacy; The hyperbaric chamber is a			

		noisy environment that disrupts communication between people inside the chamber and the outside with control panels and staff, leading to anxiety and restlessness			
Brugnolli (2023)	they report feelings of helplessness, fear, uncertainty and feeling close to the limit; fears of suffocation given by ‘wearing something that wraps around your whole head’ or ‘being enclosed in a bag’ (P 18, 5); an initial frightening impact from the feeling of not breathing and gradually the perception of benefit and relief given by their use.	Deafening noise and discomfort, dryness of the air and pressure from the suspenders and sheathing	All respondents appreciated the ‘humanity’ of HCPs and perceived the encouragement they received and the ‘reassuring presence’ as decisive in overcoming discomfort and difficulties. ‘He explained what I was up against, I get emotional thinking about it, he held my hands and very calmly explained the seriousness of it, but with great humanity. He really gave me courage’ (P 4).	Participants described helmet as a barrier between them and reality, using the terms like ‘flask’, ‘diving suit’ and one of them states: ‘you are like a fish inside an aquarium, you see others, but you can't scream and communicate’ (P 5).	Participants also gradually expressed the perceived and seen benefit of the helmet and pronation, mostly related to the ‘saturation improvement’: ‘With the helmet I could see from the monitor that my saturation was good. When they took my helmet off to eat, … it was drama. With the slightest movement, even simply swallowing, I would see the saturation drop 90–89… and I would say: -oh my God put my helmet back on before it drops any more’ (P 20).
Cervantes (2011)	Mothers described the methods of delivering oxygen as initially overwhelming and distressing. Nonetheless, with time they learned about it and became increasingly comfortable with the equipment.	Mothers spoke of the discomfort they imagined their infants felt with each type of oxygen delivery method. Mothers perceived that the intubation process was the most painful for their infants and suggested that the presence of blood after intubation was evidence of their infants’ pain. One mother described the	Information received in the NICU from staff and other parents about potential complications from oxygen delivery was a source of concern. However, most mothers explained that overall these potential negative consequences were offset by the positive benefits of oxygen therapy.	This study’s participants described that their inability to see their infants’ face or hear their voice, and not being able to hold their infants as particularly distressing aspects of various methods of oxygen delivery	All the mothers perceived that that oxygen therapy had kept their infants alive

		intubation process as an “assault” on her infant that she could not watch.			
Cirit Ekiz (2022)		During the treatment, [...] five patients [from the full-face mask group were excluded from the study] due to claustrophobia; The complaints of burning sensation and pressure in the eyes were higher in the full face mask group (p= 0.025)			
Constantin (2009)		Sophrology allowed a significant reduction in digital visual scale values in terms of reduction of discomfort (−60%), respiratory discomfort (−76%) and reduction of pain (−40%) (p < 0.001)	Restlessness and uncooperativeness resulting from pain, anxiety, and discomfort		
Dimech (2012)	Throughout the interviews feelings of entrapment were described.		Some of the patients interviewed described confusing messages that they received from health care professionals during their treatment with helmet CPAP. Patient B found that in his experience some of the nursing staff did not explain the therapy and some seemed to lack knowledge in the management of the helmet CPAP; some patients explained that they commenced or continued		all patients interviewed comment upon the environment within the helmet CPAP and its ability to help them breathe.

			treatment as they felt it was appropriate because of advice received from the health care professionals.		
Eastwood (2009)	Some [patients] felt reassured to know that they were receiving oxygen and mentioned that the flow of oxygen through the FM gave them a sense of comfort.	[patients] described device-related soreness, irritation and poor fit as contributing factors to feelings of discomfort.		Talking, eating and drinking were common activities that occurred while receiving oxygen therapy. Patients commented that the devices at times restricted these activities.	Patients considered all three low-flow oxygen therapy devices safe and effective.
Foster (2008)	Parents with babies receiving CPAP rated their satisfaction with the baby's treatment statistically significantly higher than the headbox group mean rating	Stress was perceived as at least moderate by a majority of parents; 'Baby looks in pain' and 'baby looking sad' were the most potent individual stressors for our parents within the scale 'infant behaviour and appearance'.		The highest reported stress occurred for 'separation from the baby'.	Parents of infants receiving CPAP in our study were 'extremely satisfied' with their infant's treatment compared to 'very satisfied' for the parents of infants receiving headbox oxygen.
Gebre (2022)	Initially, caregivers were anxious and doubtful about the positive outcome of bCPAP treatment, until they began to observe improvement of breathing difficulty of their children; caregivers perceived that bCPAP might be harmful to their children	[Caregivers] were concerned about the child's crying with flushed face, dry nose, and the noise of oxygen concentrator; children felt mild discomfort at the beginning of fixing the nasal prong into their nostrils	Later the clinicians explained to them how bCPAP oxygen therapy works, which made caregivers happy and relieved their anxiety.		After few hours of bCPAP treatment, all caregivers were happy, relaxed, and satisfied when they observed all signs and symptoms (e.g. fever, cough, vomiting, sneezing) were slowly disappearing and the condition of their children improved (easier breathing, sleeping, spontaneous eye opening, smiling, eating, playing), which was beyond their expectations.
Gondwe (2017)	Participants had varying reactions to bCPAP but most reported fear when they saw		Participants knew that bCPAP was treatment to provide extra oxygen to infants who were in	the caregivers reported that the tubing on bCPAP machines prevented contact	when caregivers' infants were on bCPAP, caregivers were satisfied and concluded that it

	their infants on bCPAP. They feared that their children may die or be hurt by the machine. Some participants expressed feelings such as broken heartedness, worry, disappointment, and anxiety; Most participants had prior misconceptions that oxygen delivering devices (nasal prong and bCPAP) kill.		severe respiratory distress; Many participants acknowledged that the information helped them to understand their infant's condition and bCPAP treatment. Among the participants who were told about bCPAP before it was commenced, a few participants explained that the information helped them to accept the treatment	between them and their children	was good. Some participants described it as “lifesaving”.
Hansen (2018)	Patients’ acceptance of automated oxygen delivery in our study was very high				Eight (62%) expressed very high confidence in getting the right amount of oxygen, four (31%) expressed quite a bit confidence, and one (8%) did not know. None expressed little or no confidence.
Klingenberg (2014)	The parents preferred HHHFNC as respiratory support for their infants	we found no important differences in patient comfort with HHHFNC versus NCPAP		[parents] perceived it easier to interact with their child when they were on HHHFNC	
Lucchini (2019)		patients might benefit from the use of counter-weights system thanks to better tolerance and a reduction of the pain experienced during the CPAP			
Lucchini (2024)	Common expressions were feelings of trapping, uncertainty and mental confusion, helplessness and difficult communication with	Feelings of entrapment were well-described; some participants associated entrapment with breathing difficulties. Some participants described the experience	Patients described memories of both real and surreal episodes regarding time spent with the helmet in ICU. The hallucinations were described	Separation from their loved ones; The difficulties experienced by patients in communicating with nurses and physicians were well remembered by participants	All patients expressed awareness of the importance of helmet-CPAP to improve their oxygenation and its ability to help them breathe: "it had positive effects on my

	others, as well as fear of dying.	inside the helmet as a nightmare, declaring that they almost preferred to die [than] having to put the helmet back on.	as unexpected and realistic experiences.		health (participant nr.13, male)"; "it saved my life (interview nr.16)".
McCormick (2022)		The fit around the nose was specifically called out by multiple patients, along with the mask’s tightness, restriction, and pressure on the face; The psychological impact of feeling confined and claustrophobic with the mask on their face was reported by many patients.	Patients whose provider explained what to expect with the NIV mask, the benefits of NIV, and alternatives to NIV, felt included, informed, and ready for treatment; they were more likely to “put up” with the discomfort caused by the mask.		Some patients reported that NIV helped them breathe better immediately, taking little time for the treatment to alleviate their respiratory distress; most patients realized that the treatment was needed and helped them
Peeler (2015)			Mothers described their experience of having a child in a headbox as frustrating, anxiety ridden or fearful as they did not fully understand what was happening to their child, the treatment nor illness.	The mothers stated that they were less involved with their child when he/she was receiving headbox oxygen therapy, which was described as a barrier that made them feel isolated.	Participants were unified in their perception that high-flow nasal prong oxygen therapy provided a more positive method of treating the children [than headbox oxygen].
Peterson (2023)		The most frequently reported symptoms were thirst (86, 75.4%), anxiety (68, 59.6%), tiredness (62, 54.3%), and restlessness (61, 53.5%). Most participants endorcing the symptoms of being scared and short of breath rated as severly intense (56% and 54%, respectively) and severely distressful (55% and 60%, respectively).			

Sandau (2022)	Although the robot provided a feeling of safety, it also introduced fear and a feeling of lack of safety when the therapeutic alterations were taking some time to reach the defined SpO2 range.	The patients described how they felt restricted by the nasal cannula; the patients described how the length of the robot's oxygen tube and wires constituted both a physical and a mental barrier for their activities as it made them dependent on nursing assistance to transfer to a chair, to go to the toilet, or to walk; Our findings showed that patients were willing to compromise their own safety by switching off the AOA so it would not be a "noisy burden."	The patients referred to the first few days of their hospitalization as unclear and "blurry," and did not remember that they had received oxygen treatment through a robot.	When the patients disconnected themselves from the robot (by taking off the finger clip) to eat, go to the toilet, or get dressed, an alarm would start after five minutes. The alarm would also go off if SpO2 dropped below a given range, like after a toilet visit, and then its intensity would increase with time until SpO2 was back within range. To avoid this scenario, the patients adjusted their activities of daily living by e.g. rushing through their normal routines in the toilet.	
Sandau (2023)					The choice of "Air hunger" as the most accurate descriptor was reduced during the intervention from 26 to 6 patient choices in the intervention group as opposed to a reduction from 28 to 20 patient choices in the control group.
Sessions (2020)	Almost all information participants received from the community was negative. Eighty-three per cent of mothers (45/54) explicitly, and without prompting, mentioned hearing negative opinions of supplemental oxygen therapy in the community, many using the	A majority of the mothers had no concerns about treatment. Some mothers were concerned about bruising, rashes or nasal injury.	knowledge of oxygen, and also bCPAP, was low within the participants' communities; The knowledge about bCPAP was significantly lower; most reported they had never heard of bCPAP before being educated by the study staff at the hospital. However, those who had heard of bCPAP		After treatment, the mothers were supportive of their care regardless of her child's health outcome and perceived multiple benefits of both treatments.; The most commonly discussed benefit was that the machines help children breathe.

	phrasing that 'oxygen kills'; community beliefs led mothers to feel anxious and fearful prior to treatment initiation. Additional sources of fear included lack of familiarity with the machine		reported similar negative concern that it can worsen the child's condition or kill the child.		
Stevenson (2015)	A recurrent theme was fear of oxygen, often due to a perceived association between death and recent oxygen use; reluctance to be treated with oxygen due to fear of oxygen. Participants frequently perceived it to be harmful, as many had witnessed or heard of a poor outcome following oxygen use		Many participants felt they did not know enough about oxygen and the equipment used to deliver it to make informed choices about it; Participants suggested that uptake may improve with better education about oxygen and suggested strategies such as leaflets, posters and interpersonal communication at healthcare facilities.	Other reasons for reluctance to be treated with oxygen included concerns about cost of oxygen	
Torheim (2010)	The mask treatment did at times intensify the anxiety. Some patients felt trapped as a result of the feeling of not being able to breathe; Tightening the mask as soon as it was put on made some patients afraid, as did not being able to find the bell.	Not knowing how to remove the mask and having it on for a long time without a break caused anxiety; The mask also caused some physical discomforts which could be quite painful. Pressure sores on the nose, forehead and cheeks were described	All patients interviewed stated that prior knowledge of the mask would have been advantageous.		Some were convinced that the mask 'saved their lives'.
